# Provincial export cost implications of the EU Carbon Border Adjustment Mechanism on China’s metal industry

**DOI:** 10.1016/j.isci.2025.112483

**Published:** 2025-04-17

**Authors:** Xunzhang Pan, Xin Gao, Tianpeng Wang, Wei Xiong, Tianming Shao, Meng Li, Xuan Ye, Lining Wang, Hailin Wang, Jun Pang

**Affiliations:** 1 School of Ecology & Environment, Renmin University of China, Beijing 100872, China; 2 School of Economics and Management, China University of Petroleum, Beijing 102249, China; 3 Institute of Energy, Environment, and Economy, Tsinghua University, Beijing 100084, China; 4 Institute of Nuclear and New Energy Technology, Tsinghua University, Beijing 100084, China; 5 Department of Industrial Engineering and Management, College of Engineering, Peking University, Beijing 100871, China; 6 School of Environmental Science and Engineering, Shanghai Jiao Tong University, Shanghai 200240, China; 7 Economics & Technology Research Institute, China National Petroleum Corporation, Beijing 100724, China

**Keywords:** Economics, Environmental policy

## Abstract

China’s exports of iron and steel, aluminum, and their products (ISAP) are significantly affected by the European Union (EU) Carbon Border Adjustment Mechanism (CBAM). This study introduces a modeling framework coupling a global multi-regional input-output table and the integrated assessment model GCAM-China, both of which embed Chinese provinces, to dynamically assess provincial cost implications of CBAM for China’s ISAP exports, considering China’s upcoming 2035 Nationally Determined Contributions. By 2034, direct emissions from China’s ISAP exports to the EU reach 5.4 MtCO_2_, with CBAM costs of €1,157 million. Including indirect power use raises exports to 7.4 MtCO_2_ and €1,572 million. Accounting for supply chain emissions yields 15.8 MtCO_2_ and €3,378 million. Over 65% of these costs fall on Hebei, Jiangsu, Shanxi, Liaoning, and Zhejiang. Ambitious 2035 mitigation could substantially reduce CBAM costs, with about 45% of incremental costs for accelerating electric arc furnace penetration potentially offset by avoided CBAM costs.

## Highlights

• A GMRIO-IAM modeling framework assesses provincial export cost implications of CBAM
• By 2034, China’s metal products export 5.4 MtCO_2_ to EU, with CBAM costs of €1,157 million
• Hebei, Jiangsu, Shanxi, Liaoning, and Zhejiang account for over 65% of China’ CBAM costs
• Technology progress costs of China could be partially offset by avoided CBAM costs

## Graphical abstract

**Figure:**
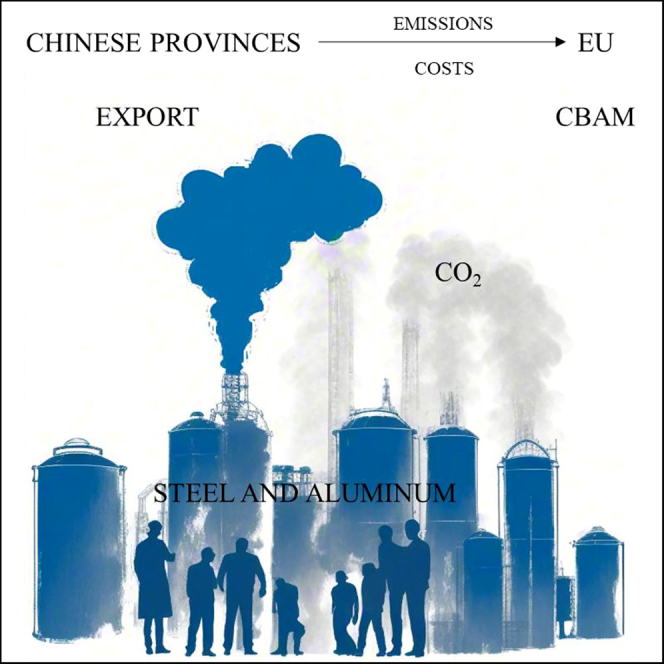

## Introduction

As countries strive to meet stringent carbon reduction targets, the potential increase in production costs raises concerns about the transfer of high-energy-consuming industries to regions with less stringent regulations, a phenomenon known as “carbon leakage.”^1^ The European Union (EU) establishes the Carbon Border Adjustment Mechanism (CBAM) to address emissions embedded in the goods exported into its customs territory, to prevent the risk of carbon leakage. At present, the EU CBAM imposes costs on the direct emissions of imported iron and steel, aluminum, hydrogen, as well as on the direct emissions and indirect emissions from power use of imported cement, electricity, and fertilizers.^2^ The EU CBAM officially entered the transition period on October 1, 2023, and is scheduled to be formally implemented in 2026.

China is currently the EU’s largest source of imports, and the embodied emissions of China’s exports to the EU show an upward trend.^3^^,^^4^^,^^5^^,^^6^^,^^7^^,^^8^ Among the goods currently covered by the EU CBAM, those related to China’s exports to the EU are iron and steel, aluminum, cement, and fertilizers. Iron and steel and aluminum covered by CBAM refer to iron and steel, aluminum, and their products (hereinafter referred to as “ISAP”; see Table S1 for the specific list). According to statistics from General Administration of Customs of the People’s Republic of China (GACC),^9^ China exported 7.43 Mt of ISAP to the EU in 2023, with export values of €13 billion, significantly higher than fertilizers and cement. The production of iron and steel and aluminum consumes a large amount of energy and results in high carbon emissions, accounting for approximately 20% of China’s current total carbon emissions.^10^^,^^11^^,^^12^ Therefore, among the goods exported from China to the EU, ISAP are most likely to be affected by CBAM. Although the current EU CBAM only imposes costs on the direct emissions from ISAP, it also requires reporting the indirect emissions from power use.^13^ In addition, future scope of emissions covered by CBAM may even be expanded to include the embodied emissions in the entire industrial chain.^14^

Assessing the cost implications of the EU CBAM on China’s ISAP exports requires a dynamic perspective, including consideration of China’s upcoming 2035 Nationally Determined Contributions (NDC), which will influence technological development in covered sectors. However, existing studies primarily rely on static assumptions regarding production intensity, export volumes, and carbon prices. For instance, although a few studies have estimated export carbon emissions associated with specific goods, including an estimate that China’s ISAP exported about 6.5 MtCO_2_ to the EU in 2019, facing CBAM costs of approximately €388 million based on a carbon price of 60 €/tCO_2_, they overlook the dynamic nature of emissions and assume that production practices will remain unchanged.^15^ Projections also indicate that the CBAM costs for China’s crude steel exported to the EU could reach about €0.72 million in 2026, based on constant production carbon intensity and an average carbon price of 81 €/tCO_2_.^16^ Moreover, existing literature tends to estimate at the national level but overlooks interprovincial variations in energy endowment and export volumes, failing to measure the impact of CBAM on different regions.^17^^,^^18^^,^^19^

Here, we constructed a new modeling framework that links a global multi-regional input-output (GMRIO) table embedding Chinese provinces with an integrated assessment model (IAM) embedding Chinese provinces, to assess carbon emissions and related CBAM costs from China’s ISAP exports to the EU (see STAR Methods). Unlike previous studies that rely on static assumptions, this framework provides a scientific approach that incorporates dynamic changes and interrelations in emission intensity, sector development, and carbon prices, in alignment with current and upcoming NDCs (see STAR Methods). With this framework, this study goes beyond national-level assessments in existing literature to account for provincial-level heterogeneity. Furthermore, this study integrates the three emission scopes potentially covered by the EU CBAM—direct carbon emissions (DCE), direct carbon emissions and indirect carbon emissions from power use (DICE), and embodied carbon emissions (ECE)—for a consistent analysis. In summary, this study extends the existing literature by contributing a comprehensive and dynamic assessment of CBAM implications for China’s ISAP exports, incorporating changes in mitigation targets and technologies, as well as provincial-level and emission-scope disparities. In the context of CBAM, the modeling framework of this study could inform similar analyses for other industries and countries and may even inspire an IO-IAM coupling paradigm for assessing environmental, economic, and policy impacts across broader fields.

## Results

### Overall impact of EU CBAM on China’s ISAP exports

**Figure 1:**
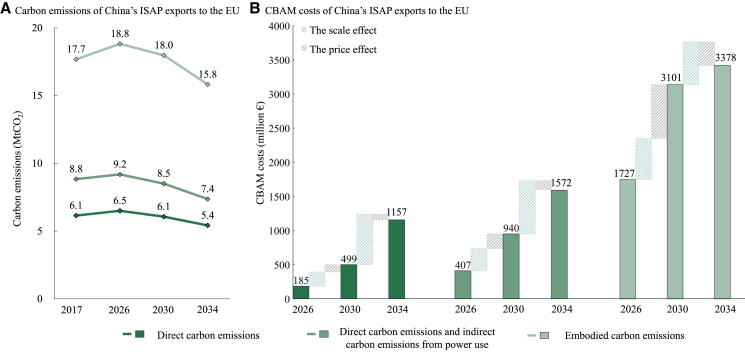
Estimation of carbon emissions and CBAM costs for China’s ISAP exports to the EU (A) Carbon emissions for China's ISAP exports to the EU. (B) CBAM costs for China's ISAP exports to the EU. This study decomposes the changes in CBAM costs into the scale effect and the price effect (see STAR Methods). The scale effect refers to changes in CBAM costs driven by variations in export carbon emissions and free allowances, whereas the price effect reflects changes due to the carbon price differential between China and the EU. This study covers 30 provinces of China, excluding Xizang (Tibet), Hong Kong, Macau, and Taiwan.

In 2017, China’s ISAP exported 6.1 MtCO_2_ of DCE to the EU (Figure 1A), which is broadly consistent with the estimates of Simola.^15^ The indirect carbon emissions from power use for China’s ISAP exports to the EU amounted to 2.7 MtCO_2_. Combined with DCE, the exported DICE reached 8.8 MtCO_2_, 1.4 times DCE alone. If CBAM expands to cover ECE along the industrial chain, carbon emissions from China’s ISAP exports increase substantially to 17.7 MtCO_2_, 2.9 times the DCE. Combining our accounting methodology with GCAM-China simulations enables estimates of future dynamics of export carbon emissions by incorporating the evolution of emission factors and changes in China-EU ISAP trade (Figure 1A). By 2026, the DCE, DICE, and ECE from China’s ISAP exports to the EU slightly increase to 6.5 MtCO_2_, 9.2 MtCO_2_, and 18.8 MtCO_2_, respectively. Until 2034, as China’s carbon emissions would have peaked, these three scopes of emissions decrease to 5.4 MtCO_2_, 7.4 MtCO_2_, and 15.8 MtCO_2_, respectively.

The implementation of CBAM imposes additional costs on China’s ISAP exports to the EU. Under their mitigation targets, EU carbon prices are modeled to reach 200 €/tCO_2_, 278 €/tCO_2_, and 309 €/tCO_2_ in 2026, 2030, and 2034, respectively, whereas China’s carbon prices reach 30 €/tCO_2_, 41 €/tCO_2_, and 95 €/tCO_2_, respectively. The scale of CBAM costs faced by China’s ISAP exports is determined by the difference in carbon prices between the EU and China, alongside export carbon emissions (Figure 1B). In 2026, China’s ISAP exports to the EU are estimated to incur costs of €185 million due to DCE. As the EU gradually reduces and eventually eliminates free carbon allowances, these costs rise to €499 million in 2030 and dramatically to €1,157 million in 2034. The CBAM costs in 2034 amount to 14% of the total export value of China’s ISAP to the EU in 2017. If CBAM also includes the indirect carbon emissions from power use for ISAP, the costs for 2026, 2030, and 2034 reach €407 million, €940 million, and €1,572 million, respectively. Should CBAM further cover ECE, these costs substantially increase to €1,727 million, €3,101 million, and €3,378 million for 2026, 2030, and 2034, respectively.

At different stages, carbon prices have varying effects on changes in CBAM costs. From 2026 to 2030, China remains in a carbon-peaking plateau phase, with an expanding carbon price differential between China and the EU. Under DCE, DICE, and ECE, the carbon price effect leads to a 33%, 40%, and 57% increase in China’s ISAP CBAM costs from 2026 to 2030, respectively. After 2030, as China implements its 2035 NDC to transition from carbon peak to carbon neutrality, the carbon price differential between China and the EU gradually decreases. The price effect becomes a driver for reducing CBAM costs from 2030 to 2034, contributing −13%, −20%, and −123% to changes in the costs under the three emission scopes, respectively. Due to the rapid phasing out of free carbon allowances by the EU, the scale effect of export carbon emissions continues to drive an increase in China’s ISAP CBAM costs from 2026 to 2034.

### Provincial-level analysis of CBAM implications

**Figure 2:**
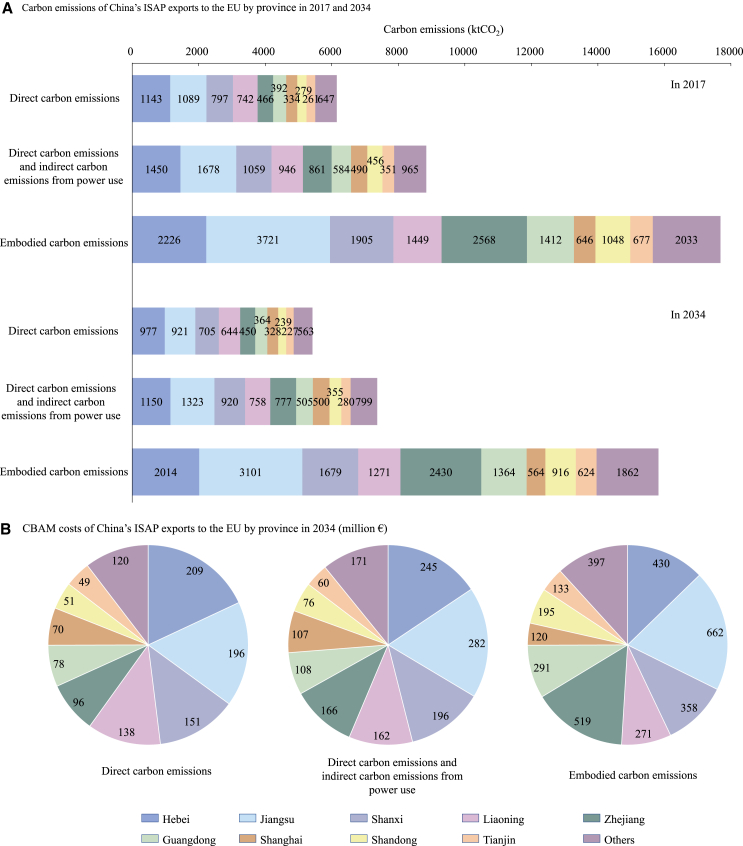
Estimation of carbon emissions and CBAM costs for China’s ISAP exports to the EU by province (A) Carbon emissions for China's ISAP exports to the EU by province. (B) CBAM costs for China's ISAP exports t the EU by province. The detailed results for “Others” can be found in Figure S3.

Due to differences in geographical location, industrial and energy structures, and economic and technological development levels, substantial variations exist in the direct carbon intensity, export volumes of ISAP, and consequent export emissions among provinces in China (Figures 2A, S1, and S2). In 2017, Hebei and Jiangsu’s ISAP exported over 1,000 ktCO_2_ of DCE to the EU, followed by the 700–800 ktCO_2_ from Shanxi and Liaoning, with emissions from Zhejiang, Guangdong, Shanghai, Shandong, and Tianjin ranging between 250 and 500 ktCO_2_. These nine provinces collectively account for 83% of China’s ISAP export volumes to the EU and 89.5% of the exported DCE in 2017. The remaining 21 provinces had DCE from ISAP exports to the EU below 130 ktCO_2_. Among different provinces, Hebei, Jiangsu, Shanxi, and Liaoning primarily export iron, steel, and aluminum to the EU, leading to higher emissions due to the energy-intensive nature of these products. In particular, Hebei, Shanxi, and Liaoning rank among the top five provinces for coal and coke consumption in the basic metals sector, resulting in the highest direct carbon intensity. By contrast, Zhejiang mainly exports manufactured products made from steel and aluminum, which have lower emissions despite its large export volumes, second only to Jiangsu (Figure 2A).

When accounting for indirect carbon emissions from power use, the provinces with the highest DCE—such as Hebei, Jiangsu, Shanxi, Liaoning, and Zhejiang—also export the largest DICE to the EU. The power structures in these provinces are predominantly coal-based, contributing to higher indirect emissions. Among them, Hebei had the highest carbon emission factor for power generation (0.91 kgCO_2_/kWh) in 2017. The DICE from ISAP exports to the EU in each of these five provinces reached 1.2 to 1.9 times their respective DCE in 2017. Notably, the indirect carbon emissions from power use for ISAP exports to the EU in Xinjiang, Inner Mongolia, Ningxia, Qinghai, Guizhou, Gansu, and Shaanxi are greater than their DCE (see Figure S3B). For example, Xinjiang’s ISAP exports are primarily aluminum (89% of its ISAP export volumes in 2017), produced mainly through electrolysis, resulting in the export DICE to the EU being 3.4 times its DCE in 2017. When expanding to ECE, due to the extensive intermediate inputs from the basic metals and power and heat supply sectors in provinces such as Hebei, Liaoning, and Shanxi, Beijing’s ECE from ISAP exports to the EU reached 10 times its DCE in 2017 (see Figure S3C). For the other 29 provinces, the ECE from ISAP exports to the EU are 1.6–6.4 times their respective DCE.

Future emissions from ISAP exported by Chinese provinces to the EU will evolve in China’s mitigation targets. Overall, the ISAP export carbon emissions of each province are projected to decrease slightly in 2034 compared to the 2017 levels (Figures 2 and S3D–S3L). Hebei, Jiangsu, Shanxi, Liaoning, Zhejiang, Guangdong, Shanghai, Shandong, and Tianjin’s ISAP continue to export the highest carbon emissions to the EU, and once CBAM is officially implemented, these provinces are projected to face substantial cost impacts. Regardless of the emission scope, in 2034, Hebei’s CBAM costs account for over 13% of China’s total, Jiangsu for over 17%, Shanxi for over 11%, and Liaoning and Zhejiang for over 8%. These nine provinces collectively account for nearly 90% of China’s total CBAM costs (Figure 2B).

### Future uncertainties: Policy, technology, and trade dynamics

The uncertainties of CBAM cost implications on China’s ISAP exports arise from following factors: the strength of future carbon mitigation targets, choices in technological development, and changes in trade trends. Mitigation targets and technological choices jointly determine China’s and the EU’s carbon prices, sectoral carbon emissions, power emission factors, and energy use in iron and steel production across Chinese provinces. In particular, the level of electric arc furnace (EAF) penetration plays a key role in shaping the carbon intensity of ISAP production.^20^ Trade trends, meanwhile, influence the scale of carbon emissions from China’s ISAP exports to the EU. To address these uncertainties, we analyzed various carbon mitigation targets for 2035, ranging from conservative to ambitious, different levels of EAF penetration, and five key trade parameters related to future sector output, export value, and export volume (see STAR Methods). This multi-angle approach provides a comprehensive understanding of how these factors contribute to the variability in China’s ISAP CBAM costs.

**Figure 3:**
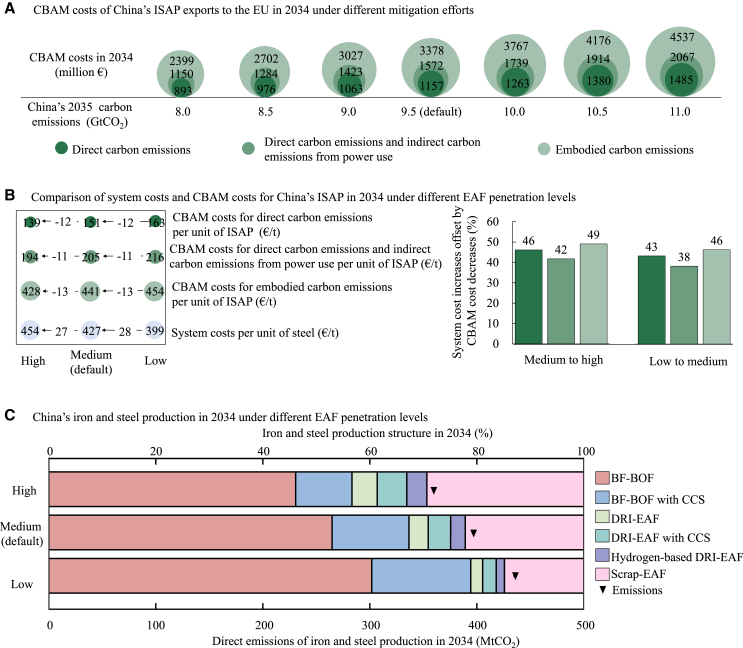
CBAM costs of China’s ISAP exports to the EU in 2034 under different mitigation targets and technology penetration In (A), the numbers, listed from bottom to top, represent the CBAM costs of China’s ISAP exports to the EU corresponding to direct carbon emissions, direct carbon emissions and indirect carbon emissions from power use, and embodied carbon emissions under different China’s 2035 carbon emissions level. In (B), “system costs” refer to China’s production costs per ton of iron and steel, including new investments and input costs for energy, scrap steel, etc., as simulated by GCAM-China for China’s iron and steel sector. In (B) and (C), China’s carbon emissions for 2035 are assumed at 9.5 GtCO_2_ (default). The results under different mitigation targets are shown in Figure S4, with the full range of cost offset ratios being 34%–56%.

Under a less ambitious mitigation target, where China reduces emissions slightly from the peak to 11 GtCO_2_ by 2035 (a 5% reduction from peak emissions), the estimated CBAM costs for China’s ISAP in 2034 would be €1,485 million for DCE, €2,067 million for DICE, and €4,537 million for ECE (Figure 3A). These represent increases of 28%, 31%, and 34%, respectively, compared to the default results reported above. For every 0.5 GtCO_2_ increase in emissions mitigation, the costs decrease by approximately €98.5 million for DCE, €152.8 million for DICE, and €56.4 million for ECE. In contrast, if China pursues an ambitious mitigation strategy, reducing emissions to 8 GtCO_2_ by 2035 (a 30% reduction), the ISAP CBAM costs for 2034 are projected to drop to €893 million for DCE, €1,150 million for DICE, and €2,399 million for ECE, representing decreases of 23%, 27%, and 29%, respectively, from the default results. The reduction in costs for DCE is smaller compared to the reductions for DICE and ECE, highlighting the challenge of decarbonizing ISAP-related industries, which are deeply embedded in China’s energy system and considered hard to abate.

Technological progress—particularly the penetration of EAF technology—also plays a crucial role in determining ISAP CBAM costs. Under high EAF penetration of this study, system costs per ton of iron and steel in China in 2034 increase by €27 compared to the default results (medium penetration) (Figure 3B). However, this increase is offset by a reduction in CBAM costs, with 46% of the incremental costs from medium to high EAF penetration that could be offset by the reduced CBAM costs. For DICE and ECE, the offset ratios are 42% and 49%, respectively. Accelerating EAF penetration not only helps lower CBAM costs but also reduces the risk of carbon “lock-in” within China’s iron and steel sector, laying a stronger foundation for achieving deep decarbonization by mid-century^21^ (Figure 3C). From medium to high EAF penetration, China’s direct emissions from iron and steel production in 2034 decrease from 397 MtCO_2_ to 360 MtCO_2_.

**Figure 4:**
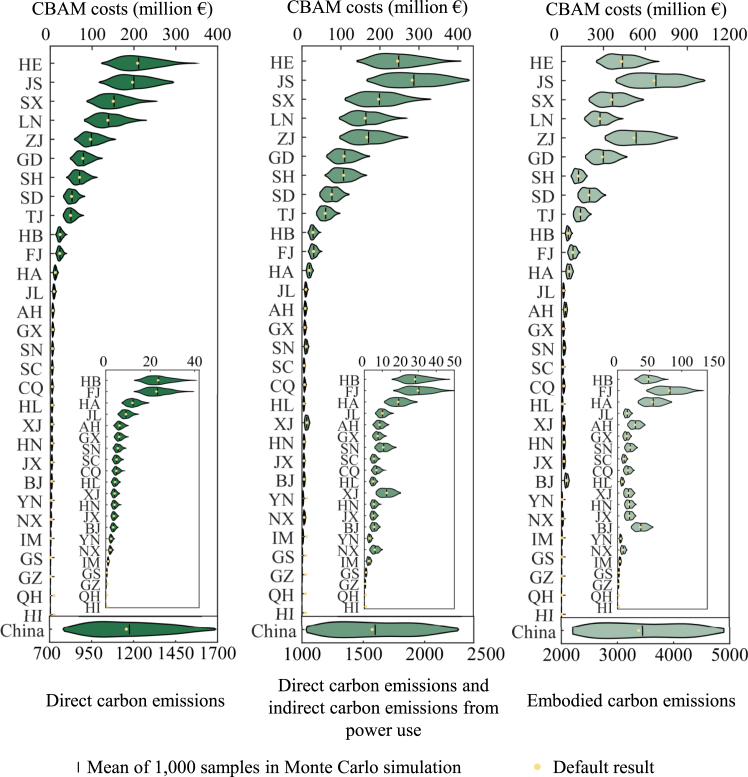
Uncertainties of CBAM costs of China’s ISAP exports to the EU in 2034 The left and right ends of the ranges represent the minimum and maximum values of the 1,000 samples in the Monte Carlo simulation, respectively. The province name codes can be found in Table S2.

To further incorporate trade uncertainty into the analysis, we employed a Monte Carlo simulation. This method involved 1,000 random samples drawn from the distribution of key trade parameters, as well as from the seven mitigation targets and three levels of EAF penetration. The results estimate the range of China’s ISAP CBAM costs in 2034, with DCE costs ranging from €773 million to €1,667 million, DICE costs from €1,024 million to €2,265 million, and ECE costs from €2,158 million to €4,946 million (Figure 4). Lower values within these ranges generally correspond to scenarios with more ambitious mitigation targets for 2035 and higher EAF penetration. At the provincial level, CBAM costs for key exporting regions—such as Hebei, Jiangsu, Shanxi, Liaoning, and Zhejiang—are most sensitive to changes in mitigation and trade parameters due to their large ISAP export volumes and high export emissions. Across the 1,000 samples, DCE costs for Hebei range from €130 million to €323 million, for Jiangsu from €115 million to €304 million, and for Shanxi from €91 million to €230 million.

## Discussion

### Contributions and discussion

**Figure 5:**
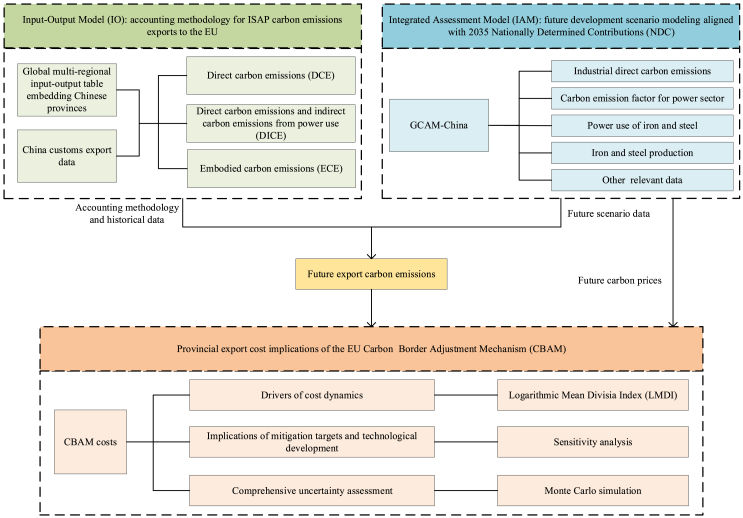
The IO-IAM modeling framework proposed in this study This study constructs an export carbon emissions accounting methodology under three different emission scopes based on the input-output model, which is first used to estimate the historical carbon emissions of China’s ISAP exports to the EU. This study then applies GCAM-China to simulate scenarios aligned with China’s NDCs, identifying the future dynamics of elements related to emissions accounting. By incorporating GCAM-China results of the energy system and carbon prices into the IO-based accounting methodology, future export carbon emissions and associated CBAM costs can be estimated. Additionally, LMDI is applied to analyze the drivers of CBAM cost changes. Sensitivity analysis and Monte Carlo simulation are used to assess the uncertainties in CBAM costs.

Existing research has largely overlooked the provincial-level implications of CBAM for China, especially considering the dynamic changes in emissions intensity, sector development, and carbon prices under the upcoming 2035 NDC. In this study, we constructed a new modeling framework by coupling a GMRIO embedding Chinese provinces with a province-level granular IAM (GCAM-China) (Figure 5). This framework dynamically quantified three scopes of carbon emissions from China’s ISAP exports to the EU at the provincial level, and assessed the resulting CBAM costs, in alignment with China’s potential 2035 mitigation targets. This IO-IAM framework could not only inform similar analyses but also provide insights into broader environmental economic issues.

Simola^15^ estimated that without free allowances, China’s ISAP CBAM costs for DCE amounted to €388 million. This is lower than the €1,157 million estimated in this study for 2034 (the year without free allowances). Simola’s study simply assumed a carbon price differential of 60 €/tCO_2_ between China and the EU, whereas GCAM-China simulated a differential of 214 €/tCO_2_ for 2034 under their mitigation targets. Combined with the decomposition analysis, this suggests that, in addition to the scale of export emissions, the carbon price differential is a key factor influencing the specific CBAM costs. The EU trading partners, including China, could pay close attention to the formulation and implementation of CBAM rules, as well as trends in EU carbon prices. Meanwhile, they could accelerate the improvement of their own carbon trading and pricing mechanisms, and promptly incorporate industries such as iron and steel and electrolytic aluminum, which are covered by CBAM. China could strive to propose ambitious mitigation targets for its 2035 NDC. This not only provides a robust start for achieving carbon neutrality before 2060 but also helps alleviate the impact of mechanisms like CBAM on exports. For example, if China could reduce its emissions to 8.0 GtCO_2_ by 2035 instead of 11.0 GtCO_2_, its ISAP CBAM costs for DCE in 2034 would decrease by 40%, for DICE by 44%, and for ECE by 47%.

China, particularly its provinces with the extensive export emissions to the EU, could view CBAM as a driver for transformational development—rather than a trade barrier—to further promote the low-carbon transformation and green upgrade of the ISAP industry. Notably, a considerable portion of the costs associated with promoting low-carbon technology penetration could be offset by the avoided CBAM costs (see Figure S4). China could guide ISAP-related enterprises to strengthen technology and process innovations, promote the elimination of outdated steel and aluminum production capacities, and accelerate the penetration of advanced production technologies such as the EAF and short-process steelmaking.

CBAM has clearly stated that it will impose costs on the indirect emissions from power use in cement and fertilizers. Given the significant increase in electrification levels in the future,^22^ China could accelerate the development of non-fossil energy and retrofit (with carbon capture and storage [CCS]) or retire coal power plants to decarbonize the power system, thereby reducing power carbon emission factors. In fact, existing studies suggest that to enable national carbon peak and carbon neutrality goals, China’s power sector peaks its carbon emissions before 2030 and achieves carbon neutrality around 2050.^23^^,^^24^ ISAP-related enterprises in China could strive to purchase green electricity directly from new energy projects such as photovoltaics and wind power to minimize indirect emissions in their production processes. In the context of CBAM and global carbon neutrality, these recommendations could also be applicable to other industries and countries.

Currently, CBAM does not cover the industrial chain of ISAP, so it primarily impacts ISAP themselves, with minimal effects on upstream and downstream goods. Existing research also confirms this, indicating that under the current good coverage, the implementation of CBAM primarily affects the basic metals sector within China’s ISAP industrial chain.^25^^,^^26^^,^^27^ China Customs export data show that the export of ISAP upstream goods from China to the EU is very small, whereas the export of downstream goods such as machinery, electrical equipment, and transportation equipment is large. Our further analysis (Figure S5) finds that if CBAM is expanded to cover these downstream machinery and equipment, China’s CBAM costs could increase substantially by tens of times, with an additional €14.7 billion for DCE and €35.5 billion for DICE in 2034. To address this underlying challenge and reduce the embodied emissions of ISAP, China could guide ISAP upstream and downstream sectors toward a green, low-carbon, and efficient development paradigm, promoting a carbon-neutral mindset throughout the entire ISAP industrial chain.

### Limitations of the study

This study still has limitations. When calculating the indirect carbon emissions from power use, we mainly considered the dynamic evolution of the carbon emission factors of power generation and the weight of power sources for each province. Including interprovincial power transmission within factories and the phenomenon of power flowing into other provinces through intermediate provinces could further improve the results. Additionally, we referred to existing studies^28^^,^^29^^,^^30^^,^^31^ to assume that the direct consumption coefficients remain unchanged until 2034. Considering that policy implementation cycles and structural adjustments typically take a relatively long time, it is unlikely that the production and market structures of most sectors in China would undergo significant changes before 2034. Therefore, in the near term, direct consumption coefficients tend to be less likely to experience significant shocks. Accurately assessing and quantifying the dynamics of these coefficients warrant further in-depth research in IO forward-looking analyses.

## Resource availability

### Lead contact

Further information and requests for resources should be directed to and will be fulfilled by the lead contact, Tianpeng Wang (wtpeng@mail.tsinghua.edu.cn).

### Materials availability

This study did not generate new unique reagents.

### Data and code availability

• Data: the data related to this paper is available on Figshare (link: https://doi.org/10.6084/m9.figshare.28892900).
• Code: the code and documentation of the GCAM-China are open source and available on GitHub (link: https://github.com/umd-cgs/gcam-china).
• Any additional information required to reanalyze the data reported in this paper is available from the lead contact upon request.

## Acknowledgments

This work is supported by 10.13039/501100012166National Key Research and Development Program of China (No. 2022YFB1903100), Project (No. 72304167 and 72174105) of the 10.13039/501100001809National Natural Science Foundation of China, Technology Development Project of China Petroleum and Chemical Corporation (No. 325089 and 322123).

## Author contributions

X.P., T.W., and H.W. conceived the study; X.P., X.G., T.W., and H.W. constructed the methodology; and X.P., X.G., and T.S. performed the analysis and created the figures. All the authors contributed to discussion and writing the manuscript.

## Declaration of interests

The authors declare no competing interests.

## STAR★Methods

### Key resources table

**Table:** 

REAGENT or RESOURCE	SOURCE	IDENTIFIER
**Deposited data**		

GMRIO table embedding Chinese provinces	China’s multi-regional input-output model: 1987–2017^32^	N/A
China’s provincial CO_2_ emission and energy inventory	Carbon Emission Accounts and Datasets (CEADs)^33^	CEADs: https://www.ceads.net.cn/data/province
China Customs export data	GACC^9^	GACC: http://stats.customs.gov.cn/
China’s provincial carbon emission factors for power generation	Chinese Academy of Environmental Planning^34^	http://www.caep.org.cn/sy/tdftzhyjzx/zxdt/202310/t20231027_1044179.shtml
China’s provincial power use and interprovincial power transmission	China Electricity Council^35^^,^^36^	N/A
Research-generated data	Authors	Figshare: https://doi.org/10.6084/m9.figshare.28892900

**Software and algorithms**		

GCAM-China	Open code	Github: https://umd-cgs.github.io/metarepo_gcam-china/index.html

### Method details

#### Overview of the modeling framework

As shown in Figure 5, the modeling framework in this study consists of a GMRIO table and an IAM (GCAM-China), both representing China at the provincial level. In this framework, the IO model is combined with official customs export data to measure historical DCE, DICE, and ECE from ISAP exports to the EU at the provincial level. GCAM-China is responsible for simulating future dynamics of key accounting elements aligned with current and upcoming NDCs, including sectoral carbon emissions, sectoral power use, power generation carbon emission factors, iron and steel production at the provincial level in China, as well as carbon prices in China and the EU. By coupling these two models, this framework provides a scientific approach to assess the dynamic changes of carbon emissions from ISAP exports to the EU and related CBAM costs by different provinces, enhancing the understanding of the cost implications of the EU CBAM.

#### Accounting methodology for export carbon emissions

This study integrates three emission scopes – DCE, DICE, and ECE – to establish a systematic accounting methodology for the carbon emissions of China’s ISAP exports to the EU. In this study, the DCE refer to the carbon emissions produced by using fossil energy (such as raw coal, coke, fuel oil, and natural gas) in the production process of ISAP. The EU CBAM regulation categorizes the covered goods into two types: simple goods and complex goods.^2^ The DCE of simple goods refer to the direct emissions from their production process, while the DCE of complex goods also include the DCE of the simple goods used that are covered by CBAM. For example, the DCE of screws include both the direct emissions from their own production and the direct emissions from the steel used in their production. Although the production of some iron and steel requires sintered ore, a precursor material covered by CBAM, this study does not include the emissions from sintered ore production due to a lack of statistical data, and the fact that the production emissions of sintered ore are much smaller than those of iron and steel. Accordingly, iron and steel and aluminum are treated as simple goods in this study, while iron and steel and aluminum products are complex goods. The indirect carbon emissions from power use in this study refer to the emissions associated with power use during the ISAP production process. The ECE in this study include all direct and indirect carbon emissions for ISAP throughout the entire process of raw material acquisition, manufacturing, processing, storage, transportation, and distribution to consumers.^7^

Due to the lack of China’s provincial-level data on the production and carbon emissions of primary aluminum, as well as of iron and steel and aluminum products, it is hard to directly account for the emissions per unit for all ISAP. This study establishes an accounting methodology based on a GMRIO table embedding Chinese provinces and China’s provincial customs export volume data. By matching the Combined Nomenclature (CN) codes of CBAM goods with China’s customs codes, and according to the China’s industrial classification for national economic activities, this study finds that all iron and steel and aluminum covered by CBAM belong to the basic metals sector in the IO table, while all iron and steel and aluminum products belong to the metal products sector (see Table S1).

##### Direct carbon emissions

The DCE of iron and steel and aluminum exported from province *p* in China to the EU ($DE_{s}^{p}$) are calculated according to Equation 1. By multiplying the direct carbon intensity of the base metals sector by the export values of this sector to the EU, the DCE of the sector’s exports to the EU are obtained, which are then allocated to iron and steel and aluminum based on customs export volume data. In Equation 1, $f_{s}^{p}$ represents the direct carbon intensity of the basic metals sector in province *p* (unit: tCO_2_/million $), $e_{s}^{p}$ represents the total export values of the basic metals sector from province *p* to the EU (unit: million $), $h_{s}^{p}$ represents the export volumes of iron and steel and aluminum covered by CBAM from province *p* to the EU (unit: t), and $H_{s}^{p}$ represents the total export volumes of the basic metals sector from province *p* to the EU (unit: t). $f_{s}^{p}$ is calculated by $C_{s}^{p}$/ $x_{s}^{p}$, where $C_{s}^{p}$ represents DCE of the basic metals sector in province *p* (unit: tCO_2_), and $x_{s}^{p}$ represents the total output/input of the basic metals sector in province *p* (unit: million $).

(Equation 1) $$DE_{s}^{p}=f_{s}^{p}e_{s}^{p}\frac{h_{s}^{p}}{H_{s}^{p}}=\frac{C_{s}^{p}}{x_{s}^{p}}e_{s}^{p}\frac{h_{s}^{p}}{H_{s}^{p}}$$

The DCE of iron and steel and aluminum products exported from province *p* in China to the EU ($DE_{c}^{p}$) are calculated according to Equation 2. As complex goods, the DCE of iron and steel and aluminum products originate both from their own production process and from the production of the iron and steel and aluminum used in them. The latter calculation relies on the direct consumption coefficients from the IO table. In Equation 2, $f_{c}^{p}$ represents the direct carbon intensity of the metal products sector in province *p*, $a_{s,c}^{q,p}$ represents the direct consumption coefficient of the metal products sector in province *p* for the basic metals sector in province *q*, $e_{c}^{p}$ represents the total export values of the metal products sector from province *p* to the EU, $h_{c}^{p}$ represents the export volumes of iron and steel and aluminum products covered by CBAM from province *p* to the EU, and $H_{c}^{p}$ represents the total export volumes of the metal products sector from province *p* to the EU. $f_{c}^{p}$ is calculated by $C_{c}^{p}$/ $x_{c}^{p}$, where $C_{c}^{p}$ represents DCE of the metal products sector in province *p*, and $x_{c}^{p}$ represents the total output/input of the metal products sector in province *p*. Finally, the DCE of the ISAP exported from province *p* to the EU ($DE^{p}$) are the sum of $DE_{s}^{p}$ and $DE_{c}^{p}$.

(Equation 2) $$DE_{c}^{p}=\left(f_{c}^{p}+\sum_{q}f_{s}^{q}a_{s,c}^{q,p}\right)e_{c}^{p}\frac{h_{c}^{p}}{H_{c}^{p}}=\left(\frac{C_{c}^{p}}{x_{c}^{p}}+\sum_{q}\frac{C_{s}^{q}}{x_{s}^{q}}a_{s,c}^{q,p}\right)e_{c}^{p}\frac{h_{c}^{p}}{H_{c}^{p}}$$

##### Direct carbon emissions and indirect carbon emissions from power use

The indirect carbon emissions from power use of iron and steel and aluminum exported from province *p* in China to the EU ($IE_{s}^{p}$) are calculated according to Equation 3. Due to power transmission among Chinese provinces, the power used by province *p* is supplied not only by itself but also by other provinces. The power carbon emission factor for each province is weighted according to the sources of power. The power carbon emission factor multiplied by the power use of the basic metals sector determines the carbon emissions from power use in that sector. In Equation 3, $f_{E}^{q}$ represents the carbon emission factor of power generation in province *q* (unit: kgCO_2_/kWh), $T^{q,p}$ represents the amount of power transmitted from province *q* to province *p* (unit: kWh), $D^{p}$ represents the total power use of province *p* (unit: kWh), and $EC_{s}^{p}$ represents the power use of the basic metals sector in province *p* (unit: kWh).

(Equation 3) $$IE_{s}^{p}=\frac{\sum_{q}f_{E}^{q}\left(T^{q,p}/D^{p}\right)EC_{s}^{p}}{x_{s}^{p}}e_{s}^{p}\frac{h_{s}^{p}}{H_{s}^{p}}$$

The indirect carbon emissions from power use of iron and steel and aluminum products exported from province *p* in China to the EU ($IE_{c}^{p}$) are calculated according to Equation 4. Similarly, $IE_{c}^{p}$ includes the carbon emissions associated with the power used in the production of iron and steel and aluminum products themselves, as well as the power used in producing the iron and steel and aluminum used in them. The calculation of the latter relies on the direct consumption coefficients of the IO table. In Equation 4, $EC_{c}^{p}$ represents the power use of the metal products sector in province *p*. Finally, the indirect carbon emissions from power use of the ISAP exported from province *p* to the EU ($IE^{p}$) are the sum of $IE_{s}^{p}$ and $IE_{c}^{p}$. The DICE of the ISAP exported from province *p* to the EU are the sum of $DE^{p}$ and $IE^{p}$.

(Equation 4) $$IE_{c}^{p}=\sum_{q}\left[\frac{f_{E}^{q}\left(T^{q,p}/D^{p}\right)EC_{c}^{p}}{x_{c}^{p}}+\frac{\sum_{r}f_{E}^{r}\left(T^{r,q}/D^{q}\right)EC_{s}^{q}}{x_{s}^{q}}a_{s,c}^{q,p}\right]e_{c}^{p}\frac{h_{c}^{p}}{H_{c}^{p}}$$

##### Embodied carbon emissions

The ECE of iron and steel and aluminum exported from province *p* in China to the EU ($EE_{s}^{p}$) are calculated according to Equation 5. The ECE calculation relies on the total requirement coefficients (Leontief inverse matrix) from the IO table. In Equation 5, $EE_{i,s}^{p}$ represents $EE_{s}^{p}$ originated from the input of sector *i*, $f_{i}^{q}$ represents the direct carbon intensity of sector *i* in province *q*, and $l_{i,s}^{q,p}$ presents the total requirement coefficient of the basic metals sector in province *p* for sector *i* in province *q*. $f_{i}^{q}$ is calculated by $C_{i}^{q}$/ $x_{i}^{q}$, where $C_{i}^{q}$ represents DCE of sector *i* in province *q*, and $x_{i}^{q}$ represents the total output/input of sector *i* in province *q*.

(Equation 5) $$EE_{s}^{p}=\sum_{i}EE_{i,s}^{p}=\sum_{i}\sum_{q}f_{i}^{q}l_{i,s}^{q,p}e_{s}^{p}\frac{h_{s}^{p}}{H_{s}^{p}}=\sum_{i}\sum_{q}\frac{C_{i}^{q}}{x_{i}^{q}}l_{i,s}^{q,p}e_{s}^{p}\frac{h_{s}^{p}}{H_{s}^{p}}$$

The ECE of iron and steel and aluminum products exported from province *p* in China to the EU ($EE_{c}^{p}$) are calculated according to Equation 6, where $EE_{i,c}^{p}$ represents $EE_{c}^{p}$ originated from the input of sector *i*, and $l_{i,c}^{q,p}$ represents the total requirement coefficient of the metal products sector in province *p* for sector *i* in province *q*. Finally, the ECE of the ISAP exported from province *p* to the EU ($EE^{p}$) are the sum of $EE_{s}^{p}$ and $EE_{c}^{p}$.

(Equation 6) $$EE_{c}^{p}=\sum_{i}EE_{i,c}^{p}=\sum_{i}\sum_{q}f_{i}^{q}l_{i,c}^{q,p}e_{c}^{p}\frac{h_{c}^{p}}{H_{c}^{p}}=\sum_{i}\sum_{q}\frac{C_{i}^{q}}{x_{i}^{q}}l_{i,c}^{q,p}e_{c}^{p}\frac{h_{c}^{p}}{H_{c}^{p}}$$

For the variables in Equations 1, 2, 3, 4, 5, and 6, the data sources are shown in Table S3.

##### GMRIO

The GMRIO table embedding Chinese provinces used in this study is from Li et al.,^32^ which is developed based on the 2017 intercountry IO table from the Organisation for Economic Co-operation and Development (OECD) (OECD: https://www.oecd.org/sti/ind/inter-country-input-output-tables.htm) and the 2017 Chinese interprovincial IO table from the Development Research Center of the State Council of China. Currently, the IO tables related to China mainly include the GMRIO tables published by WIOD/OECD/GTAP/EORA/EXIOBASE (and other organizations), the provincial IO tables of China published by CEADs, and the GMRIO table embedding Chinese provinces used in this study. In WIOD/OECD/GTAP/EORA/EXIOBASE GMRIO tables, China is treated as a single region without detailed provincial division. The CEADs provincial IO table of China depicts interprovincial trade but aggregates international trade, lacking detailed information on each province’s trade with individual countries and regions such as the EU. The GMRIO table embedding Chinese provinces used in this study divides mainland China into 31 provinces, and divides other countries in the world into 96 countries and regions, including the EU 27 members. Compared with other IO tables, this table links each Chinese province with every country worldwide, detailing the scale and characteristics of trade between different Chinese provinces and various countries globally. This table has been used in recent studies to account for CO_2_ emissions and value-added flows between Chinese provinces and other countries,^37^ as well as to analyze the division of China’s value chain from a global perspective.^38^ The framework of this table is illustrated in Figure S6.

Since the latest year for this table is 2017, this study calculates the historical carbon emissions of ISAP exports for the year 2017. Due to the lack of data for Xizang in the CEADs provincial carbon emission inventory, this study assesses 30 provinces of China, excluding Xizang, Hong Kong, Macau, and Taiwan.

#### Estimation of CBAM costs

When imposing costs on the carbon emissions of imported goods, CBAM considers the free allowances allocated to domestic homogeneous goods by the EU and the carbon costs already paid for the goods in the country of origin.^2^ In December 2022, the European Parliament and EU countries reached an agreement on the reform of the EU Emission Trading Scheme (ETS), determining that starting in 2026, the EU ETS’s free allowances will be gradually reduced from the 2021–2025 benchmark, with a 2.5% reduction in 2026, a 48.5% reduction in 2030, and complete abolition in 2034.^39^ The EU CBAM regulation stipulates that the implementation of CBAM will proceed in parallel with the abolition of free allowances under the EU ETS. This means that the free allowances for goods covered by CBAM will also be gradually abolished in accordance with the timeline of the EU ETS.^2^ Considering the implementation process of CBAM, this study focuses on the cost implications of CBAM until 2034. According to the Ministry of Ecology and Environment of China,^40^ cement, iron and steel, and electrolytic aluminum will soon be formally included in China’s national ETS. Referring to Li et al.^41^ and Ren et al.,^42^ this study calculates the CBAM costs for exporting ISAP from province *p* in China to the EU between 2026 and 2034 according to Equation 7. In this equation, $P^{\text{EU}}$ represents the EU CBAM certificate price (€/tCO_2_), $P^{\text{CHN}}$ represents the carbon price in China (€/tCO_2_), $E^{p}$ represents the carbon emissions of ISAP exported from province *p* in China to the EU (e.g., $DE^{p}$, $DE^{p}$ + $IE^{p}$, and $EE^{p}$), $q_{is}$ represents the free allowances for iron and steel and products (unit: tCO_2_/t), $q_{al}$ represents the free allowances for aluminum and products (unit: tCO_2_/t), $h_{is}^{p}$ represents the export volumes of iron and steel and products from province *p* to the EU, and $h_{al}^{p}$ represents the export volumes of aluminum and products from province *p* to the EU. The raw materials for iron and steel products are primarily iron and steel, while the raw materials for and aluminum products are primarily aluminum. Therefore, this study assumes that the free allowances for iron and steel products are the same as those for iron and steel, and that the free allowances for aluminum products are the same as those for aluminum. In 2034 (and thereafter), as the free allowances are completely phased out, Equation 7 simplifies to Equation 8.

(Equation 7) $$T^{p}=\left(P^{\text{EU}}-P^{\text{CHN}}\right)\left(E^{p}-\left(q_{is}h_{is}^{p}+q_{al}h_{al}^{p}\right)\right)$$

(Equation 8) $$T^{p}=\left(P^{\text{EU}}-P^{\text{CHN}}\right)E^{p}$$

Currently, in the benchmark for free allowances determined by the EU for 2021-2025,^43^ coke (0.217 tCO_2_/t), sintered ore (0.157 tCO_2_/t), and hot metal (1.288 tCO_2_/t) are related to iron and steel and products, while aluminum (1.464 tCO_2_/t) is related to aluminum and products. Referring to Zhang et al.,^16^ this study uses the hot metal allowances in the benchmark as the free allowances for iron and steel and products for 2021–2025, and uses the aluminum allowances as the free allowances for aluminum and products. From 2026 to 2034, $q_{is}$ and $q_{al}$ in Equation 7 are calculated based on the reduction rate of the EU ETS free allowances.

##### GCAM-China

The future values for other variables in Equations 7 and 8, including $E^{p}$, $P^{\text{CHN}}$ and $P^{\text{EU}}$, are estimated based on GCAM-China (v6). GCAM-China is developed based on GCAM (Global Change Analysis Model; Github: http://jgcri.github.io/gcam-doc/index.html), a well-known IAM developed by Pacific Northwest National Laboratory. The core of GCAM is the energy system, which provides a detailed representation of the entire energy chain, covering energy extraction (including various fossil and non-fossil energy sources), transformation (including power generation, oil refining, gas processing, hydrogen production, and heating), and end-use (including industry, buildings, and transportation). GCAM incorporates a wide range of technologies and options across different sectors and processes of the energy system. These technologies compete for market shares based primarily on cost through a probabilistic logit equation, as shown in Equation 9, where $share_{j}$ is the share of technology *j*, $sw_{j}$ is the share weight of technology *j*, *γ* is the logit exponent, and $m_{j}$ is the levelized cost of technology *j*. GCAM uses exogenous share weights to reflect policy or public preferences for particular technology choices, as well as to enable the gradual phasing-in of new technologies.^44^

(Equation 9) $$share_{j}=\frac{sw_{j}m_{j}^{\gamma }}{\sum_{j}sw_{j}m_{j}^{\gamma }}$$

GCAM divides the world into 32 geopolitical regions, with China as a separate region. Within the GCAM framework, GCAM-China introduces finer spatial details and more specific features for the China region (Github: https://umd-cgs.github.io/metarepo_gcam-china/metarepo.html). It disaggregates China’s energy-economic system into 31 provincial-level sub-regions. In other words, GCAM-China remains a global model and features a disaggregation of the China region into its provinces. In GCAM-China, electricity generation and energy end-use are modeled at the provincial level. Renewable energy resources and carbon storage potential are also provincial-specific. GCAM-China has been increasingly applied in recent research.^45^^,^^46^

##### Assumption of upcoming NDC

According to the time frame under the Paris Agreement, countries are currently formulating a new round of NDCs for 2035. For China, the upcoming 2035 NDC serves as a critical milestone in guiding the transition of its socioeconomic and energy systems from carbon peak to carbon neutrality. The default scenario of this study assumes that China’s energy system emissions reach 11.5 GtCO_2_ in 2030 and decrease to 9.5 GtCO_2_ in 2035. This emissions level is approximately positioned in the middle of the 2035 emissions ranges reported in existing studies^23^^,^^47^^,^^48^^,^^49^^,^^50^ on China’s carbon neutrality pathways, and represents a 15–20% reduction from peak emissions. As a partial equilibrium model (with a five-year time step), GCAM-China dynamic-recursively solves for carbon prices (which are used as the values of $P^{\text{CHN}}$ in this study) until the sum of provincial carbon emissions strictly meets the assumed carbon constraints for China. Since GCAM-China is a global model that also include the EU region, this study uses it to simulate the EU climate pledges and obtain the corresponding carbon prices as the values of $P^{\text{EU}}$. The EU’s emissions pathway is set to achieve a 55% reduction in 2030 and a 90% reduction in 2040 from 1990 levels.

##### Estimation of future export carbon emissions

This study links GCAM-China future simulations with the IO-based accounting methodology to derive future carbon emissions from China’s provincial ISAP exports to the EU. The sector matching between the GMRIO table and the GCAM-China energy system is provided in Table S4. GCAM-China provides a detailed provincial-scale characterization of China’s iron and steel sector, including six specific technologies: blast furnace-basic oxygen furnace (BF-BOF), BF-BOF with CCS, direct reduced iron-EAF (DRI-EAF), DRI-EAF with CCS, hydrogen-based DRI-EAF, and scrap-EAF. Due to the lack of sufficient provincial-level data, GCAM-China has not yet explicitly represented the non-ferrous metal (aluminum) sector or the metal products sector. Therefore, in this study, both the basic metals sector and the metal products sector in the GMRIO table are matched to the iron and steel sector in GCAM-China.

By using GCAM-China to simulate emission reduction scenarios aligned with current and assumed upcoming NDCs, future values of accounting-related elements under these scenarios are obtained, such as future sectoral carbon emissions, power generation carbon emission factors, and iron and steel production and power use at the provincial level in China (as shown in Tables S2, S5, and S6). Due to minor discrepancies between GCAM-China historical data and data sources such as CEADs (Table S3), this study uses the change rates simulated by GCAM-China to estimate the DCE of various sectors and the power use of the iron and steel sector in each province in the future. For example, the future DCE for sector *i* in province *p* ($C_{i}^{p}\left(t\right)$) are calculated using Equation 10 where $C_{i}^{p}\left(0\right)$ represents DCE of sector *i* in province *p* in 2017 from the CEADs emission inventory, while $GC_{i}^{p}\left(t\right)$ and $GC_{i}^{p}\left(0\right)$ represent DCE of sector *i* in province *p* in future year *t* and 2017 in GCAM-China simulations, respectively (GCAM-China results for 2017, 2026 and 2034 are obtained through interpolation).

(Equation 10) $$C_{i}^{p}\left(t\right)=C_{i}^{p}\left(0\right)\frac{GC_{i}^{p}\left(t\right)}{GC_{i}^{p}\left(0\right)}$$

In the future, there is uncertainty surrounding trade between China and the EU. GCAM-China is a technology-oriented model, and its representation of the economy and trade is limited. In the default analysis of this study, the proportion of iron and steel and aluminum in the export volumes of the basic metals sector to the EU ($h_{s}^{p}$/ $H_{s}^{p}$), the proportion of iron and steel and aluminum products in the export volumes of the metal products sector to the EU ($h_{c}^{p}$/ $H_{c}^{p}$), the proportion of the basic metals sector’s export value to the EU in the sector’s total output $\left(e_{s}^{p}/x_{s}^{p}\right)$, and the proportion of the metal products sector’s export value to the EU in the sector’s total output $\left(e_{c}^{p}/x_{c}^{p}\right)$ all remain at 2017 levels until 2034 for each province. We further refer to a recent study^51^ to include the dynamic changes in the weight of power sources for each province. At this point, we can estimate $DE_{s}^{p}\left(t\right)$ and $IE_{s}^{p}\left(t\right)$. Due to the difficulties in compiling future GMRIO tables and the likelihood that iron and steel production in China is still dominated by blast furnaces and converters in the short term, in order to estimate $DE_{c}^{p}\left(t\right)$, $IE_{c}^{p}\left(t\right)$, $EE_{s}^{p}\left(t\right)$, and $EE_{c}^{p}\left(t\right)$, this study maintains the values from the current GMRIO table^28^^,^^29^^,^^30^^,^^31^ for the total requirement coefficients of the basic metals sector ($l_{i,s}^{q,p}$) and the direct consumption ($a_{s,c}^{q,p}$) and total requirement coefficients of the metal products sector ($l_{i,c}^{q,p}$) in each province until 2034.

This study estimates the future total output of each sector in each province of China based on the GDP growth and changes in industrial structure. The future total output of sector *i* in province *p* ($x_{i}^{p}\left(t\right)$) is calculated according to Equation 11. In this equation, $x_{i}^{p}\left(0\right)$ represents the total output of sector *i* in province *p* in 2017 in the GMRIO table; $GDP^{p}\left(t\right)$ and $GDP^{p}\left(0\right)$ represent the GDP of province *p* in future year *t* and 2017 in GCAM-China (the growth rate follows the SSP2 scenario), respectively; and $R_{I}^{p}\left(t\right)$ and $R_{I}^{p}\left(0\right)$ represent the share of value-added of industry *I* (primary, secondary, or tertiary; depending on the industry to which sector *i* belongs) in province *p* relative to GDP in future year *t* (sourced from Li et al.,^52^ as shown in Figure S7) and 2017, respectively. Finally, to estimate the specific CBAM costs, the change rates of each province’s export volumes of iron and steel and products ($h_{is}^{p}$) and aluminum and products ($h_{al}^{p}$) to the EU before 2034, are considered according to the change rates of iron and steel production in each province, as simulated by GCAM-China.

(Equation 11) $$x_{i}^{p}\left(t\right)=x_{i}^{p}\left(0\right)\frac{GDP^{p}\left(t\right)R_{I}^{p}\left(t\right)}{GDP^{p}\left(0\right)R_{I}^{p}\left(0\right)}$$

##### Decomposition of changes in CBAM costs

This study applies the LMDI^53^ to decompose the changes in CBAM costs into two drivers – the scale effect and the price effect, as shown in Equation 12. In this equation, $T^{p}\left(t\right)$ and $T^{p}\left(t_{0}\right)$ represent the ISAP CBAM costs of province *p* in China in year *t* and year $t_{0}$, respectively; the scale effect $\Delta T_{CE}^{p}$ refers to changes in CBAM costs driven by changes in the difference between export carbon emissions and free allowances; and the price effect $\Delta T_{CP}^{p}$ refers to changes in CBAM costs driven by changes in the carbon price differential between China and the EU. The specific contributions of these two effects are quantified in Equations 13 and 14, where $CE^{p}\left(t\right)$ and $CE^{p}\left(t_{0}\right)$ represent the carbon emissions (deducting free allowances) of ISAP exported from province *p* in China to the EU in year *t* and year $t_{0}$, respectively (the second term in Equations 7 and 8)); and $CP^{p}\left(t\right)$ and $CP^{p}\left(t_{0}\right)$ represent the carbon price differential between China and the EU in year *t* and year $t_{0}$, respectively (the first term in Equations 7 and 8).

(Equation 12) $$\Delta T^{p}=T^{p}\left(t\right)-T^{p}\left(t_{0}\right)=\Delta T_{CE}^{p}+\Delta T_{CP}^{p}$$

(Equation 13) $$\Delta T_{CE}^{p}=\frac{T^{p}\left(t\right)-T^{p}\left(t_{0}\right)}{\ln\left(T^{p}\left(t\right)/T^{p}\left(t_{0}\right)\right)}\ln\frac{CE^{p}\left(t\right)}{CE^{p}\left(t_{0}\right)}$$

(Equation 14) $$\Delta T_{CP}^{p}=\frac{T^{p}\left(t\right)-T^{p}\left(t_{0}\right)}{\ln\left(T^{p}\left(t\right)/T^{p}\left(t_{0}\right)\right)}\ln\frac{CP^{p}\left(t\right)}{CP^{p}\left(t_{0}\right)}$$

##### Sensitivity analysis and Monte Carlo simulation

This study considers mitigation targets, technological development, and trade uncertainties to further understand the cost implications of CBAM on China’s ISAP exports to the EU. First, based on the default assumption that China’s emissions reach 9.5 GtCO_2_ in 2035, this study additionally considers six different mitigation targets to reflect the uncertainty of China’s upcoming 2035 NDC, with 2035 carbon emissions ranging from 8.0 to 11.0 GtCO_2_ (in increments of 0.5 GtCO_2_). This study conducts the sensitivity analysis on China’s 2035 mitigation targets while maintaining the default assumptions on technological development and trade.

As mentioned earlier, the share weights in GCAM-China (Equation 9) can represent the market’s willingness or preference to adopt a particular technology. Higher share weights implicitly indicate a stronger market or policy willingness to promote technology penetration. Based on the default share weights for EAF in GCAM-China (considered as ‘medium’ penetration), this study adjusts the share weights upward and downward (see Figure S8) to represent ‘high’ and ‘low’ penetration, respectively, reflecting the uncertainty in ISAP technological development. Theoretically, the faster the EAF penetration, the higher the electrification level of the ISAP industry, and the lower the DCE. The sensitivity analysis on technological development in this study is conducted while maintaining the default trade assumptions.

Regarding trade uncertainty, this study considers five key trade-related parameters, including future sector output ($x_{i}^{p}$), the proportion of the basic metals sector’s export value to the EU in the sector’s total output $\left(e_{s}^{p}/x_{s}^{p}\right)$, the proportion of the metal products sector’s export value to the EU in the sector’s total output $\left(e_{c}^{p}/x_{c}^{p}\right)$, the proportion of iron and steel and aluminum covered by CBAM in the export volumes of the basic metals sector to the EU $\left(h_{s}^{p}/H_{s}^{p}\right)$, and the proportion of iron and steel and aluminum products covered by CBAM in the export volumes of the metal products sector to the EU $\left(h_{c}^{p}/H_{c}^{p}\right)$. Ultimately, this study integrates the uncertainties in these three dimensions to conduct a Monte Carlo simulation^54^ of CBAM costs and performs 1,000 samples. Existing studies typically apply the uniform distribution to construct distributions for demand and emission targets in Monte Carlo simulations.^55^^,^^56^^,^^57^ In each sampling, this study selects one of the seven 2035 mitigation targets and one of the three EAF penetration levels with equal probability, and generates stochastic factors for each of the five trade parameters according to a uniform distribution with a mean of 1. The specific distribution design is summarized in Table S7. These stochastic factors are generated as vectors to allow for differentiated changes across provinces, with the values of the trade parameters being the product of their default values and the corresponding stochastic factors.

### Quantification and statistical analysis

The Monte Carlo simulation was conducted using MATLAB. Figures 1, 2, and S6–S8 were created using Microsoft PowerPoint. Figures 3, 4, S4, and S5 were created using MATLAB and Microsoft PowerPoint. Figures S1–S3 were created using ArcMap. Details of the Monte Carlo simulation can be found in method details.

### Additional resources

There are no additional resources to include in our study.
