# Supplementary material for: Provincial export cost implications of the EU Carbon Border Adjustment Mechanism on China’s metal industry
Source: iScience. 2025 Apr 17;28(5):112483. doi: 10.1016/j.isci.2025.112483 (PMC12146617; doi:10.1016/j.isci.2025.112483)
Supplement: Document S1. Figures S1–S8 and Tables S1–S7 [file mmc1.pdf]

iScience, Volume *28*

Supplemental information

Provincial export cost implications of the EU Carbon Border Adjustment Mechanism on China's metal industry

Xunzhang Pan, Xin Gao, Tianpeng Wang, Wei Xiong, Tianming Shao, Meng Li, Xuan Ye, Lining Wang, Hailin Wang, and Jun Pang

SUPPLEMENTAL FIGURES

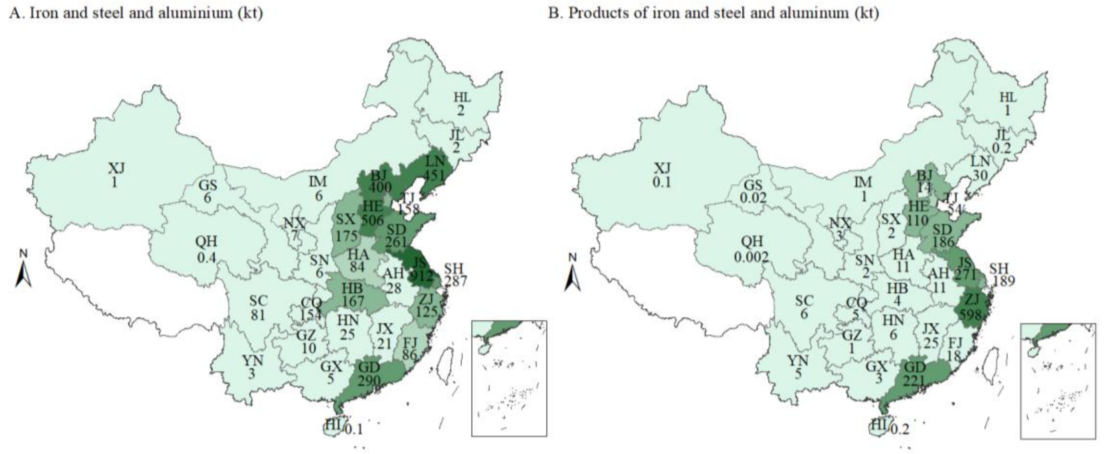

Figure S1. China's ISAP export volumes to the EU by province in 2017, related to RESULTS.

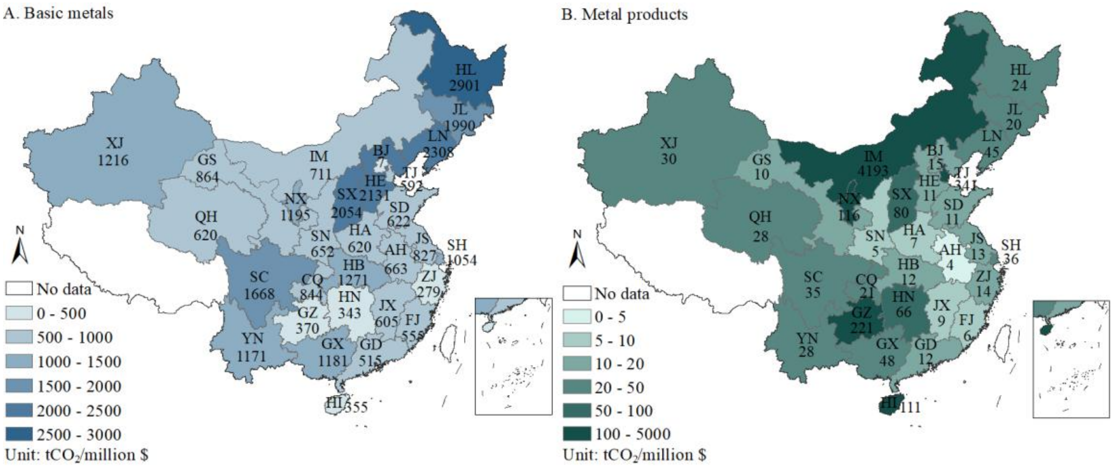

Figure S2. Direct carbon intensity of the basic metals sector and metal products sector in Chinese provinces in 2017, related to RESULTS.

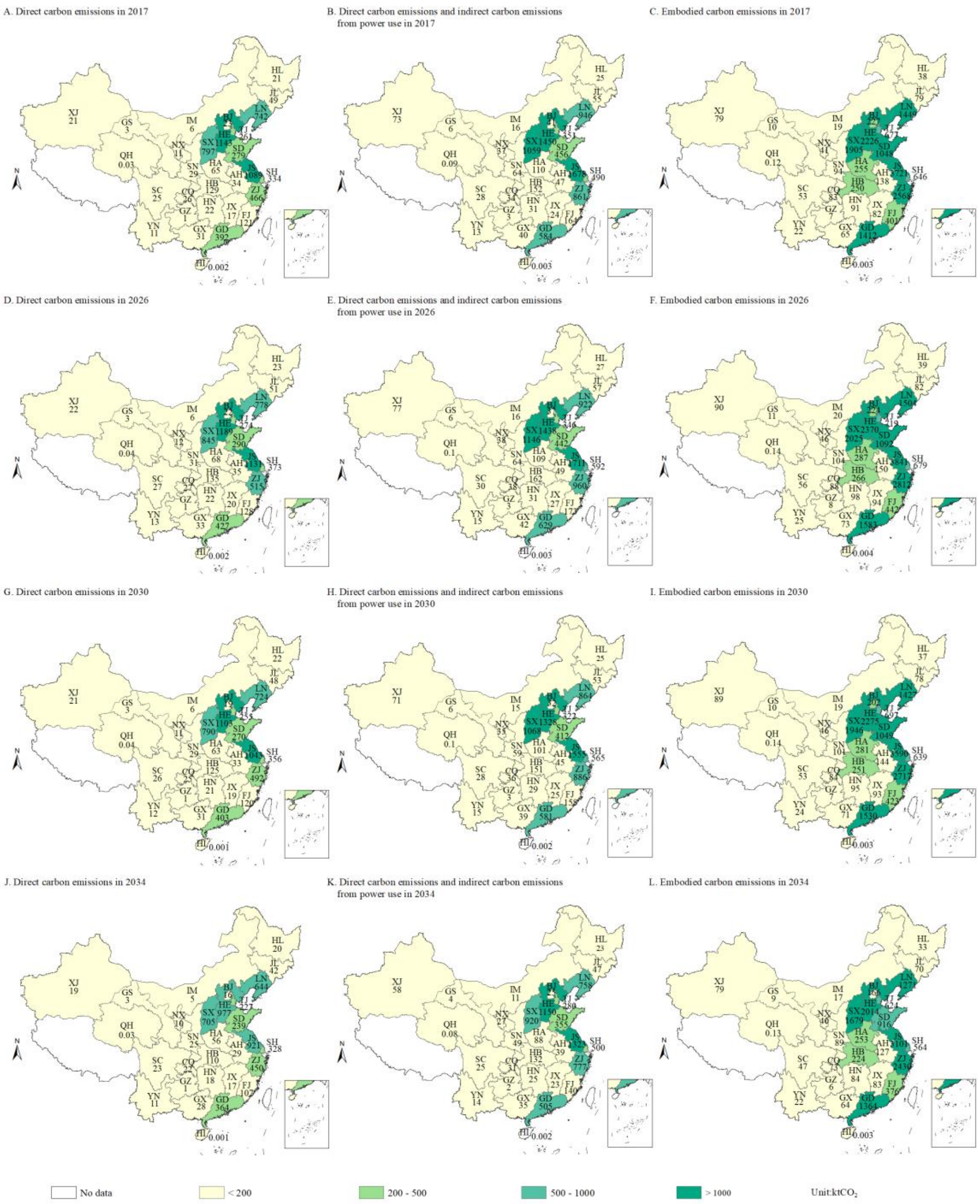

Figure S3. Dynamics of carbon emissions from Chinas' ISAP exports to the EU by province from 2017 to 2034, related to Figure 2 and RESULTS.

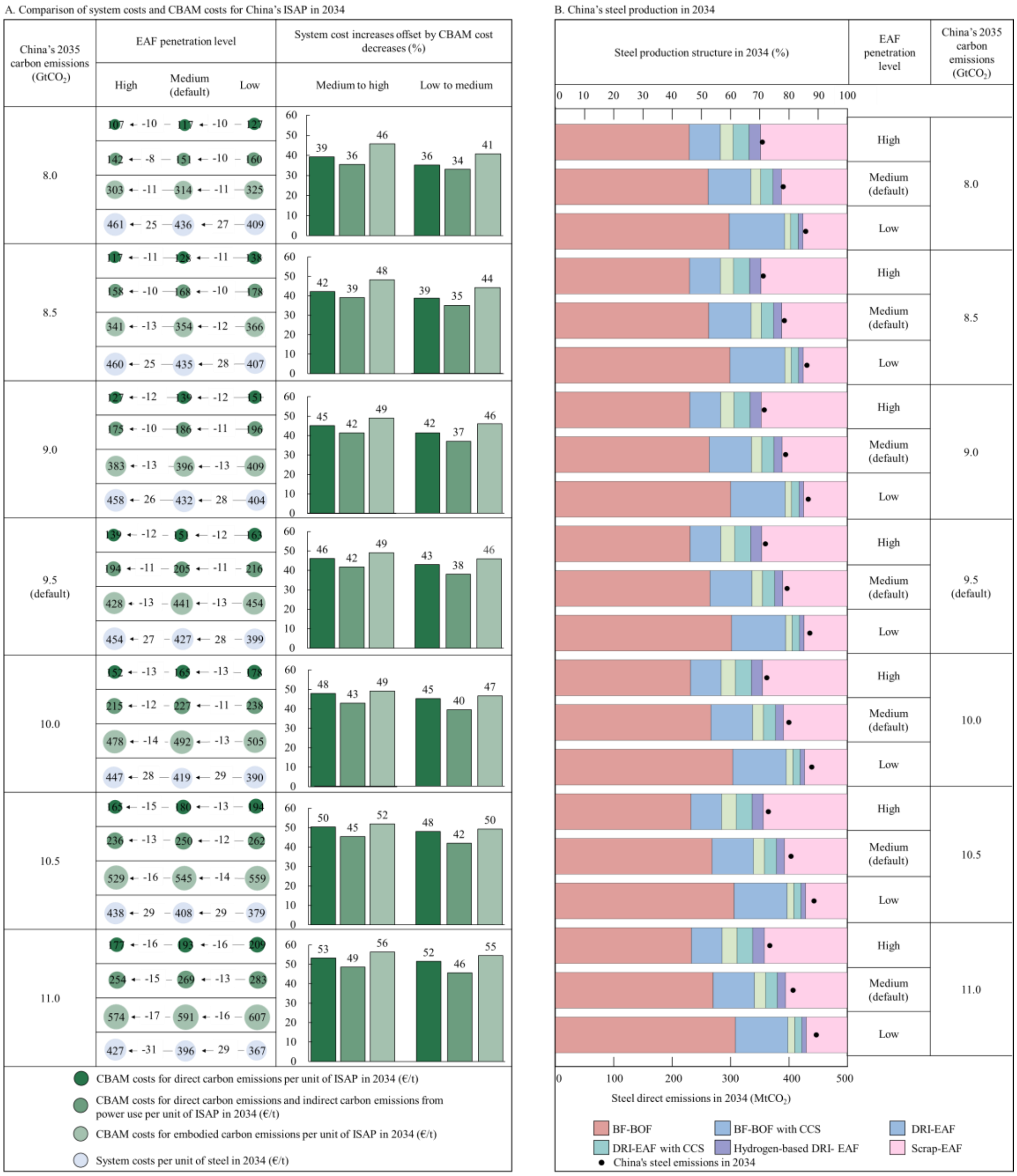

Figure S4. Comparison of system costs and CBAM costs (A) and production structure (B) for China's ISAP in 2034, related to Figure 3 and DISCUSSION and CONCLUSIONS. In (A), 'system costs' refer to the costs per ton of iron and steel, including new investments and input costs for energy, scrap steel, etc., as simulated by GCAM-China for China's iron and steel sector.

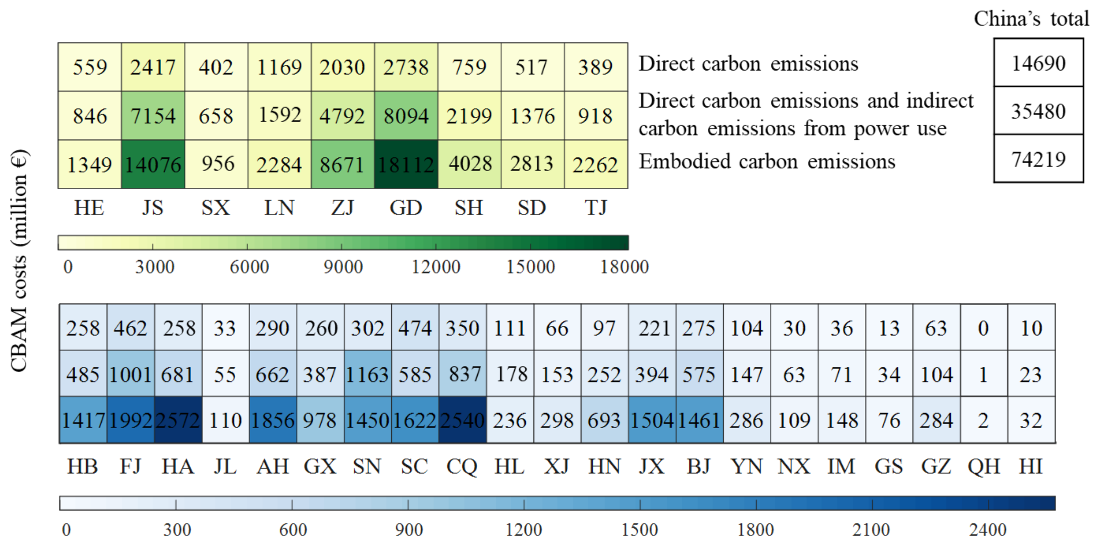

Figure S5. CBAM costs of China's machinery and equipment exports to the EU in 2034, related to DISCUSSION and CONCLUSIONS. This figure is created under the assumption that ISAP downstream machinery and equipment are covered by CBAM. For convenience in estimation, we assessed China's exports to the EU in four sectors: 'manufacture of communication equipment, computers and other electronic equipment; manufacture of measuring instruments', 'manufacture of electrical machinery and equipment', 'manufacture of general and special purpose machinery', and 'manufacture of transport equipment' (sectors 12~15 in Table S4). The results are calculated under the default scenario following the methods proposed in the main text. The province name codes can be found in Table S2.

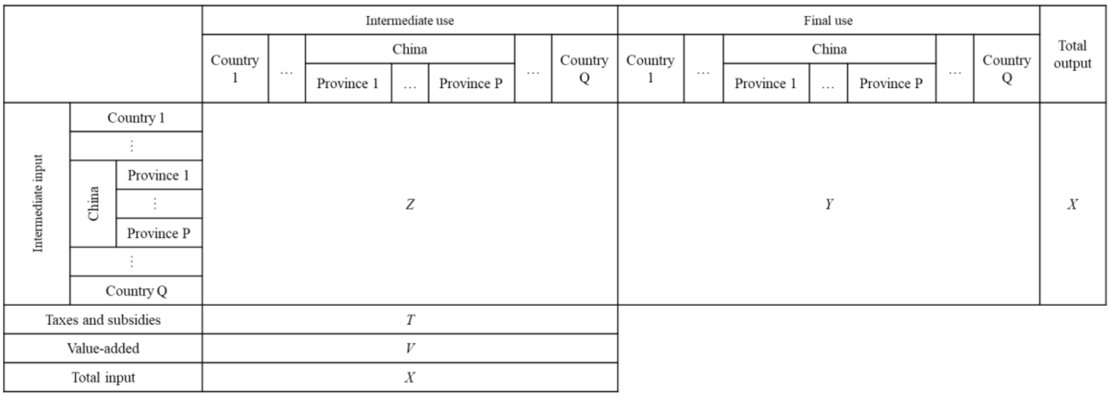

Figure S6. The structure of the global multi-regional input-output table embedding Chinese provinces, related to STAR METHODS.

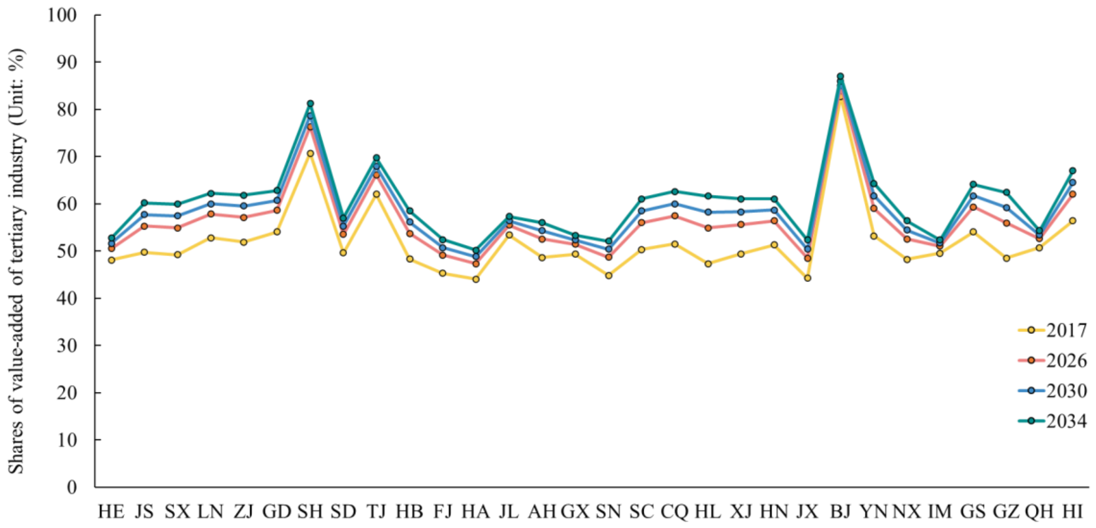

Figure S7. The share of value-added of the tertiary industry relative to GDP in Chinese provinces, related to STAR METHODS. Future shares are sourced from Li et al [S1]. The province name codes can be found in Table S2.

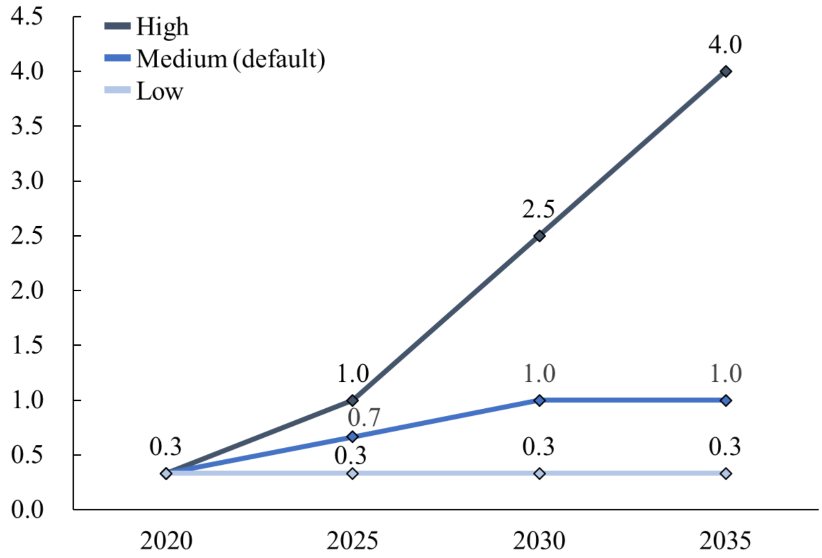

Figure S8. The share weight settings for representing different levels of EAF penetration in GCAM-China, related to STAR METHODS. The ‘medium’ share weights are the default settings in GCAM-China. This study first sets the ‘low’ share weights to remain unchanged from 2020 to 2035. GCAM-China simulations show that under this ‘low’ penetration (with China’s energy system emissions at 9.5 GtCO2 in 2035), China’s EAF production of iron and steel in 2035 is about 35% lower than under ‘medium’ penetration. Subsequently, the ‘high’ share weights are set so that China’s EAF production of iron and steel in 2035 is about 35% higher than under ‘medium’ penetration.

SUPPLEMENTAL TABLES

Table S1. Iron and steel and aluminum covered by the CBAM, related to INTRODUCTION and STAR METHODS.

	CN code	HS code	IO Industry
Iron and steel	72 – Iron and steel<br>Except:	72	Basic metals
	72022 – Ferro-silicon	720211、720229	
	72023000 – Ferro-silico-manganese	72023000	
	72025000 – Ferro-silico-chromium	72025000	
	72027000 – Ferro-molybdenum	72027000	
	72028000 – Ferro-tungsten and ferro-silico-tungsten	72028000	
	72029100 – Ferro-titanium and ferro-silico-titanium	72029100	
	72029200 – Ferro-vanadium	720292	
	72029300 – Ferro-niobium	72029300	
	720299 – Other:<br>72029910 – Ferro-phosphorus<br>72029930 – Ferro-silico-magnesium<br>72029980 – Other		
	7204 – Ferrous waste and scrap; remelting scrap ingots and steel	7204	
	26011200 – Agglomerated iron ores and concentrates, other than roasted iron pyrites	26011200	
	7301 – Sheet piling of iron or steel, whether or not drilled, punched or made from assembled elements; welded angles, shapes and sections, of iron or steel	7301	
	7302 – Railway or tramway track construction material of iron or steel, the following: rails, check-rails and rack rails, switch blades, crossing frogs, point rods and other crossing pieces, sleepers (cross-ties), fish- plates, chairs, chair wedges, sole plates (base plates), rail clips, bedplates, ties and other material specialized for jointing or fixing rails	7302	
	730300 – Tubes, pipes and hollow profiles, of cast iron	730300	
	7304 – Tubes, pipes and hollow profiles, seamless, of iron (other than cast iron) or steel	7304	
	7305 – Other tubes and pipes (for example, welded, riveted or similarly closed), having circular cross-sections, the external diameter of which exceeds 406,4 mm, of iron or steel	7305	
	7306 – Other tubes, pipes and hollow profiles (for example, open seam or welded, riveted or similarly closed), of iron or steel	7306	
	7307 – Tube or pipe fittings (for example, couplings, elbows, sleeves), of iron or steel	7307	
	7308 – Structures (excluding prefabricated buildings of heading 9406) and parts of structures (for example, bridges and bridge-sections, lock- gates, towers, lattice masts, roofs, roofing frameworks, doors and windows and their frames and thresholds for doors, shutters, balustrades, pillars and columns), of iron or steel; plates, rods, angles, shapes, sections, tubes and the like, prepared for use in structures, of iron or steel	7308	Metal products
	730900 – Reservoirs, tanks, vats and similar containers for any material (other than compressed or liquefied gas), of iron or steel, of a capacity exceeding 300 l,	730900	

	whether or not lined or heat-insulated, but not fitted with mechanical or thermal equipment		
	7310 – Tanks, casks, drums, cans, boxes and similar containers, for any material (other than compressed or liquefied gas), of iron or steel, of a capacity not exceeding 300 l, whether or not lined or heat-insulated, but not fitted with mechanical or thermal equipment	7310	
	731100 – Containers for compressed or liquefied gas, of iron or steel	731100	
	7318 – Screws, bolts, nuts, coach screws, screw hooks, rivets, cotters, cotter pins, washers (including spring washers) and similar articles, of iron or steel	7318	
	7326 – Other articles of iron or steel	7326	
Aluminum	7601 – Unwrought aluminum	7601	Basic metals
	7603 – Aluminum powders and flakes	7603	
	7604 – Aluminum bars, rods and profiles	7604	
	7605 – Aluminum wire	7605	
	7606 – Aluminum plates, sheets and strip, of a thickness exceeding 0,2 mm	7606	
	7607 – Aluminum foil (whether or not printed or backed with paper, paper board, plastics or similar backing materials) of a thickness (excluding any backing) not exceeding 0,2 mm	7607	
	7608 – Aluminum tubes and pipes	7608	
	76090000 – Aluminum tube or pipe fittings (for example, couplings, elbows, sleeves)	76090000	Metal products
	7610 – Aluminum structures (excluding prefabricated buildings of heading 9406) and parts of structures (for example, bridges and bridge sections, towers, lattice masts, roofs, roofing frameworks, doors and windows and their frames and thresholds for doors, balustrades, pillars and columns); aluminum plates, rods, profiles, tubes and the like, prepared for use in structures	7612	
	76110000 – Aluminum reservoirs, tanks, vats and similar containers, for any material (other than compressed or liquefied gas), of a capacity exceeding 300 litres, whether or not lined or heat insulated, but not fitted with mechanical or thermal equipment	7613	
	7612 – Aluminum casks, drums, cans, boxes and similar containers (including rigid or collapsible tubular containers), for any material (other than compressed or liquefied gas), of a capacity not exceeding 300 litres, whether or not lined or heat insulated, but not fitted with mechanical or thermal equipment	7614	
	76130000 – Aluminum containers for compressed or liquefied gas	7616	
	7614 – Stranded wire, cables, plaited bands and the like, of aluminum, not electrically insulated	7610	
	7616 – Other articles of aluminum	76110000	

Table S2. Iron and steel production simulated by GCAM-China (Unit: Mt), related to Figure 4 and STAR METHODS.

Province		2015	2020	2025	2030	2035
HE	Hebei	188.3	247.5	269.5	279.8	278.7
JS	Jiangsu	110	144.5	157.4	163.4	162.7
SX	Shanxi	38.5	50.6	55.1	57.2	56.9
LN	Liaoning	60.7	79.8	86.9	90.2	89.8
ZJ	Zhejiang	15.9	21	22.8	23.7	23.6
GD	Guangdong	17.6	23.1	25.2	26.2	26.1
SH	Shanghai	17.8	23.4	25.5	26.5	26.4
SD	Shandong	66.2	87	94.7	98.4	97.9
TJ	Tianjin	20.7	27.2	29.6	30.7	30.6
HB	Hubei	29.2	38.4	41.8	43.4	43.2
FJ	Fujian	15.9	20.8	22.7	23.6	23.5
HA	Hainan	29	38.1	41.5	43.1	42.9
JL	Jilin	10.7	14	15.3	15.9	15.8
AH	Anhui	25.1	32.9	35.9	37.2	37.1
GX	Guangxi	21.5	28.2	30.7	31.9	31.8
SN	Shaanxi	10.3	13.5	14.7	15.3	15.2
SC	Sichuan	19.5	25.6	27.9	28.9	28.8
CQ	Chongqing	6.9	9.1	9.9	10.2	10.2
HL	Heilongjiang	4.2	5.5	6	6.2	6.2
XJ	Xinjiang	7.4	9.7	10.6	11	10.9
HN	Hunan	18.5	24.3	26.5	27.5	27.4
JX	Jiangxi	22.1	29.1	31.6	32.9	32.7
BJ	Beijing	0	0	0	0	0
YN	Yunnan	14.2	18.6	20.3	21.1	21
NX	Ningxia	1.8	2.4	2.6	2.7	2.7
IM	Inner Mongolia	17.4	22.8	24.8	25.8	25.7
GS	Gansu	8.5	11.2	12.2	12.7	12.6
GZ	Guizhou	4.7	6.1	6.7	6.9	6.9
QH	Qinghai	1.2	1.6	1.7	1.8	1.8
HI	Hainan	0.2	0.3	0.3	0.4	0.4

Table S3. Data sources of variables, related to STAR METHODS.

Variables	Sources	Notes
x、*e*、*a*、*l*	The GMRIO table embedding Chinese provinces [S2]	This study uses the direct carbon intensity of the basic metals sector and metal products sector in the IO table (as shown in Figure S3) to calculate the carbon emissions of ISAP. Between 2013 and 2023, iron and steel and aluminum accounted for approximately 97% of China's total metal production [S4]
C	China provincial CO_2 emission inventory from the Carbon Emission Accounts and Datasets (CEADs) [S3]	
h、*H*	General Administration of Customs of the People's Republic of China [S5]	
f_E	Chinese Academy of Environmental Planning [S6]	The Chinese Academy of Environmental Planning released the provincial-level carbon emission factors for power generation for 2018. This study estimates these provincial-level factors for 2017 by comparing China's national power carbon emission factors for 2018 and 2017 [S7]. That is, a province's carbon emission factor for power generation for 2017 is equal to its 2018 factor divided by the national rate of change
T、*D*	China Electricity Council [S8,S9] and Li et al.[S10]	
EC	China provincial energy inventory from CEADs [S3]	As mentioned above, the results are calculated based on the basic metals sector and metal products sector

Table S4. Sector matching between GCAM-China and the global multi-regional input-output table embedding Chinese provinces, related to STAR METHODS.

GMRIO table	CGAM-China
1. Agriculture, Forestry, Animal Husbandry and Fishery	Agriculture
2. Mining and washing of coal; Extraction of petroleum and natural gas<br>3. Mining and processing of metal ores and nonmetal and other ores	Mining
4. Food and tobacco processing<br>5. Textile sector; Manufacture of leather, fur, feather and related products<br>6. Processing of timber and furniture; Manufacture of paper, printing and articles for culture, education and sport activity, Other manufacturing and waste resources	Other industries
7. Processing of petroleum, coking, processing of nuclear fuel	Coke<br>Oil refining
8. Manufacture of chemical products	Chemical
9. Manufacture of non -metallic mineral products	Cement
10. Basic metals<br>11. Metal products	Iron and steel
12.Manufacture of communication equipment, computers and other electronic equipment; Manufacture of measuring instruments<br>13. Manufacture of electrical machinery and equipment<br>14. Manufacture of general and special purpose machinery<br>15. Manufacture of transport equipment	Other industries
16. Production and distribution of electric power, heat power and gas	Electricity and heat
17. Production and distribution of tap water	Other industries
18. Construction	Construction
19. Wholesale and retail trades<br>20. Accommodation and catering	Commercial buildings
21. Transport, storage, and postal services	Transportation
22. Culture, sports and recreation<br>23. Information transmission, software and information technology services<br>24. Finance<br>25. Real Estate<br>26. Research and experimental development; integrated technical services<br>27. Leasing and business services; water, environment and public facilities management<br>28. Residential Services, Repairs and Other Services; Public Administration, Social Security and Social Organizations<br>29. Education<br>30. Health and social work	Commercial buildings

Table S5. Power use in the iron and steel sector simulated by GCAM-China (Unit: TWh), related to STAR METHODS.

Province		2015	2020	2025	2030	2035
HE	Hebei	147.9	198.5	218.1	224	220.1
JS	Jiangsu	83.8	112.8	124.2	127.7	125.6
SX	Shanxi	24.4	34.2	38.4	40.3	40.5
LN	Liaoning	46.5	62.8	69.3	71.4	70.4
ZJ	Zhejiang	12.1	16.2	17.8	18.2	18
GD	Guangdong	13.1	17.7	19.5	20.1	20
SH	Shanghai	13	17.5	19.4	20.1	20.1
SD	Shandong	51.3	69.2	76.1	78.4	77.2
TJ	Tianjin	15.9	21.5	23.7	24.4	24.1
HB	Hubei	22.3	29.9	32.9	33.7	32.9
FJ	Fujian	12.4	16.5	18.1	18.4	18.1
HA	Hainan	21.9	29.4	32.4	33.3	32.6
JL	Jilin	8.2	11.1	12.2	12.6	12.4
AH	Anhui	19.6	26.2	28.7	29.4	28.7
GX	Guangxi	16.8	22.3	24.5	25	24.6
SN	Shaanxi	8	10.7	11.8	12.2	12
SC	Sichuan	14.2	19.3	21.4	22.1	21.9
CQ	Chongqing	5.2	7	7.7	7.9	7.8
HL	Heilongjiang	3	4.1	4.6	4.8	4.8
XJ	Xinjiang	5.7	7.7	8.5	8.8	8.7
HN	Hunan	14.2	19	20.9	21.4	20.9
JX	Jiangxi	17.3	22.9	25	25.5	24.9
BJ	Beijing	0	0	0	0	0
YN	Yunnan	2.4	4.6	5.9	6.9	7.7
NX	Ningxia	1.4	1.9	2.1	2.2	2.1
IM	Inner Mongolia	13.6	18.4	20.2	20.8	20.5
GS	Gansu	6.6	8.9	9.8	10.1	10
GZ	Guizhou	3.5	4.7	5.2	5.4	5.4
QH	Qinghai	0.8	1.1	1.3	1.3	1.4
HI	Hainan	0.2	0.3	0.3	0.3	0.3

Table S6. Carbon emissions in the iron and steel sector simulated by GCAM-China (Unit: MtCO2), related to STAR METHODS.

Province		2015	2020	2025	2030	2035
HE	Hebei	109.9	122.5	121.3	110.4	94.4
JS	Jiangsu	45.1	51.6	51.9	48.1	42.2
SX	Shanxi	22.2	25.3	25.2	23.2	20.1
LN	Liaoning	33.9	38.1	37.9	34.7	29.9
ZJ	Zhejiang	8.7	10	10.1	9.3	8.1
GD	Guangdong	9.3	10.8	10.9	10.2	9
SH	Shanghai	8	9.4	9.6	9.1	8.2
SD	Shandong	37.8	42.3	42	38.3	32.9
TJ	Tianjin	11.6	13	12.9	11.9	10.2
HB	Hubei	16.2	18.1	18	16.4	14
FJ	Fujian	9.3	10.6	10.6	9.7	8.4
HA	Hainan	15.6	17.6	17.5	16	13.8
JL	Jilin	6	6.7	6.7	6.1	5.3
AH	Anhui	14.5	16.2	16	14.5	12.4
GX	Guangxi	12.5	14.2	14.2	13.1	11.4
SN	Shaanxi	5.9	6.6	6.5	6	5.1
SC	Sichuan	9.7	11.1	11.2	10.4	9.1
CQ	Chongqing	3.7	4.2	4.2	3.8	3.3
HL	Heilongjiang	2	2.3	2.3	2.2	1.9
XJ	Xinjiang	4.2	4.7	4.7	4.3	3.7
HN	Hunan	10.3	11.5	11.4	10.4	8.9
JX	Jiangxi	6.7	7.9	8.1	7.6	6.8
BJ	Beijing	0	0	0	0	0
YN	Yunnan	8.1	9.7	10	9.6	8.8
NX	Ningxia	1.1	1.2	1.2	1.1	0.9
IM	Inner Mongolia	10.2	11.3	11.2	10.2	8.8
GS	Gansu	4.9	5.4	5.4	4.9	4.2
GZ	Guizhou	2.5	2.8	2.8	2.6	2.3
QH	Qinghai	0.5	0.6	0.6	0.6	0.5
HI	Hainan	0.1	0.2	0.2	0.1	0.1

Table S7. Parameter distribution design, related to STAR METHODS.

Dimension	Parameter	Distribution	Range
Mitigation targets	China's 2035 emissions	Discrete Uniform	{8.0, 8.5, 9.0, 9.5, 10.0, 10.5, 11.0}
Technological development	EAF penetration level	Discrete Uniform	{low, medium, high}
Trade	x_i^p , $\frac{e_s^p}{x_s^p}$, $\frac{e_c^p}{x_c^p}$, $\frac{h_s^p}{H_s^p}$, $\frac{h_c^p}{H_c^p}$	Continuous Uniform	(0.9, 1.1) * default value

SUPPLEMENTAL REFERENCES

[S1] Li, N., Chen, W., and Zhang, Q. (2020). Development of China TIMES-30P model and its application to model China's provincial low carbon transformation. Energ. Econ. 92, 104955. https://doi.org/10.1016/j.eneco.2020.104955.

[S2] Li, S., He, J., Zhu, F., Zhang Z., Pan, C., et al. (2023). China's multi-regional input-output model: 1987-2017. (Economic Science Press).

[S3] Shan, Y., Huang, Q., Guan, D., and Hubacek, K. (2020). China CO_2 emission accounts 2016–2017. Sci. Data. 7(1), 54. https://doi.org/10.1038/s41597-020-0393-y.

[S4] National Bureau of Statistics. National data. (2023). https://data.stats.gov.cn/easyquery.htm.

[S5] General Administration of Customs of the People's Republic of China. (2023). Customs Statistics Online Query Platform. http://stats.customs.gov.cn/.

[S6] Chinese Academy of Environmental Planning. China regional power grids carbon dioxide emission factors. (2023). http://www.caep.org.cn/sy/tdftzhyjzx/zxdt/202310/t20231027_1044179.shtml.

[S7] China Electricity Council. China Power Industry Annual Development Report 2018. (2018). (China Market Press).

[S8] China Electricity Council. Compilation of statistical data of power industry 2017.

[S9] China Electricity Council. China Power Industry Annual Development Report 2017. (2018). (China Market Press).

[S10] Li, W., Yang, M., Long, R., He, Z., Zhang, L., and Chen, F. (2021). Assessment of greenhouse gasses and air pollutant emissions embodied in cross-province electricity trade in China. Resour. Conserv. Recy. 171, 105623. https://doi.org/10.1016/j.resconrec.2021.105623.
